# Sex-specific associations between serum lipid levels and cognitive performance in older adults: results from a cross-sectional real-world study

**DOI:** 10.1007/s40520-025-02976-y

**Published:** 2025-03-01

**Authors:** Virginia Boccardi, Francesca Mancinetti, Anna Giulia Guazzarini, Ilenia Murasecco, Francesco Melis, Patrizia Bastiani, Michela Scamosci, Roberta Cecchetti, Patrizia Mecocci

**Affiliations:** 1 Division of Gerontology and Geriatrics, Department of Medicine and Surgery, University of Perugia, Piazzale Gambuli 1, 06132 Perugia, Italy; 2 Division of Clinical Geriatrics, Department of Neurobiology, Care Sciences and Society, Karolinska Institutet, Stockholm, Sweden

**Keywords:** Aging, Cognition, Dyslipidemia,, Dementia, Sex

## Abstract

**Aim:**

Dyslipidemia and cognitive decline are prevalent in older adults, with their incidence increasing with age. However, the relationship between serum lipid levels and cognitive dysfunction in geriatrics remains unclear, potentially influenced by sex differences.

**Methods:**

This study evaluated serum lipid levels and cognitive functions in older adults using a large battery of neuropsychological tests. Dementia was staged with the Clinical Dementia Rating (CDR), classifying participants as cognitively healthy (CDR 0), mildly impaired (CDR 0.5), or with dementia (CDR ≥ 1).

**Results:**

The study involved 1283 participants aged over 65 (466 men, 817 women; mean age 79.79 ± 5.93 years). Women had lower education levels, reduced autonomy in activities of daily living (ADL), but greater independence in instrumental ADL. Additionally, women exhibited lower glucose but higher levels of total cholesterol (TC), high-density lipoprotein (HDL-C), and low-density lipoprotein (LDL-C) compared to men. Subjects with CDR ≥ 1 had significantly poorer cognitive scores than those with CDR 0 or 0.5. No associations were found between lipid levels and cognition in the CDR 0 group. In men with CDR 0, HDL-C positively correlated with ACE-R Fluency. In the CDR 0.5 group, TC and HDL-C were linked to better cognitive performance. For CDR ≥ 1, TC and HDL-C were associated with improved cognition in women but linked to cognitive decline in men.

**Conclusion:**

Elevated late-life cholesterol may protect cognitive function in healthy individuals and those with mild impairment, with a sex-specific impact in dementia, beneficial for women but detrimental for men.

## Introduction

Cognitive impairment, which includes both mild cognitive impairment (MCI) and dementia, is on the rise as the population ages and represents a significant public health issue [1]. Alzheimer’s disease (AD), the predominant form of dementia in the older population, is recognized as a complex, progressive neurodegenerative disorder that leads to a gradual decline in all cognitive functions [2]. At present, there are few available treatments for dementia; thus, identifying potential predictors of cognitive decline represents the key to counteract such a disease. The pathogenetic mechanisms leading to AD are still unknown, but acquired causes, including vascular risk factors, increase the risk along with aging [3]. Among them, lipid levels have been extensively studied for their potential association with the disorder. Lipid metabolism plays a significant role in neuronal development, synaptic plasticity, and brain function. Serum lipids are comprised of various elements, including total cholesterol (TC), triglycerides (TG), high-density lipoprotein cholesterol (HDL-C), and low-density lipoprotein cholesterol (LDL-C). Overall, cholesterol is involved in the metabolic breakdown of amyloid precursor protein in the brain, which is critical in the onset of dementia [4–6]. Thus, it is possible to hypothesize that lipid balance disturbances may be crucial in the onset and development of cognitive disorders. Prior research has investigated the relationship between serum lipid levels and cognitive impairment in older adults, yet findings have been varied and sometimes contradictory.

Some research indeed indicates that high levels of total cholesterol and/or lipoproteins during middle age might pose a risk for later cognitive decline and dementia as individuals age [7]. Another study, instead, have found that low total cholesterol levels in old persons (aged 65 and above) could negatively affect cognitive functions. This association is evident in poorer memory and slower processing speeds, and it is linked with an increased risk of dementia [8]. These results may vary due to variations in target populations, including the stage of cognitive decline, age, and sex. Thus, studying a representative cohort of older persons cognitively healthy, affected by MCI or dementia stratified by sex could provide more precise insights into the potential connections between serum lipids and cognitive impairment.

## Methods

This retrospective study utilized data from the GeriCo study (Geriatric Cognitive Evaluation, https://gericoev.eu), a clinical-based project at the University of Perugia’s Gerontology and Geriatrics division, focused on cognitive impairment and dementia in older adults.

The general inclusion criteria were as follows:

### Healthy controls

Age and education adjusted MMSE score ≥ 27; no active neurological or psychiatric disorders; no ongoing medical problems or related treatments interfering with cognitive functions; a normal neurological exam; no psychoactive medications; the ability to live and function independently in the community.

### MCI

MCI is classified into two types: amnestic MCI (aMCI), characterized by poor episodic memory, and non-amnestic MCI (naMCI), marked by deficits in other cognitive domains like executive function, language, or visuospatial abilities. Considering that aMCI is highly associated with progression to AD [9], in this study, we included only this category of subjects. aMCI was diagnosed according to Petersen’s criteria [10].

### Alzheimer’s dementia

Dementia was diagnosed according to standard research criteria [11] by a combination of clinical and neuropsychological evaluation and brain imaging.

Between January 2016 and July 2023, a total of 2500 subjects were evaluated in our memory clinic for cognitive decline, and according to inclusion and exclusion criteria, 1283 subjects were included in the study. All participants recruited provided informed consent, and the study adhered to the Declaration of Helsinki and was approved by the Regional Ethical Committee (Prot. N. CE-1065/24 del 24/07/2024).

### Multidimensional and cognitive performance assessment

Clinical information was obtained by history and multidimensional evaluation. Data collection included an interview concerning demographics, cognitive and functional status, and pharmacological therapy. An informant-based rating of functional status was carried out using the Activity of Daily Living (ADL) and the Instrumental Activity of Daily Living (IADL) scales. Expert neuropsychologists assessed participants through a neuropsychological battery. The Mini-Mental State Examination [12] and the Addenbrooke’s Cognitive Examination-Revised (ACE-R) [13] were used as screening tests. The ACER domains include Attention and Orientation (ACER-AO), which assess alertness and awareness of time, place, and person; Memory (ACER-M), which evaluates short-term and episodic recall through word recall and recognition tasks; Fluency (ACER-F), which tests verbal fluency by requiring the patient to generate words within a category or starting with a specific letter, helping detect executive dysfunction; Language (ACER-L), which examines naming, comprehension, repetition, reading, and writing abilities to assess language impairments; and Visuospatial Abilities (ACER-V), which measure spatial recognition and manipulation through tasks like clock drawing and cube copying. Next, neuropsychological tests (Table S1) were administered to patients to assess different cognitive functions in more detail, as previously described [14]. Attention was measured using an erasure task (Matrices) and the Trail Making Test A (TMT-A). Several aspects of memory were tested with (1) the Digit Span forward test for the ability to retain visuospatial information for a short period of time; (2) the Digit Span backward test d as a measure of the central executive of working memory; (3) the Rey Auditory Verbal Learning Test Immediate (RAVLT-I) and Delayed recall (RAVLT-D) as a measure of learning and verbal memory; (4) the Prose Memory test by Babcock story recall as a measure of memory for structured verbal information (5) the Corsi span tasks as measures for verbal and visuo-spatial short-term memory. For the assessment of language skills, the Letter Fluency Test (FAS) and Categories Fluency Test were used to measure verbal fluency involving both language skills and executive functions. The Raven’s Test was used as a measure of fluid intelligence and logical reasoning. Details of administration procedures, Italian normative data for score adjustment for age and education, and normality cutoff scores are available [15]. The Clinical Dementia Rating (CDR) was used to stage dementia. It assesses cognitive and functional decline in multiple domains: memory, orientation, judgment and problem-solving skills, community relations, home and hobbies, and personal care [16]. Three groups were distinguished: cognitively healthy subjects (CDR 0), mild cognitive impairment subjects (CDR 0.5), and subjects with dementia (CDR ≥ 1).

### Clinical and biochemical variable assessment

Anthropometric determinations (weight, height) were measured using standard techniques. Body Mass Index (BMI) was calculated as weight in kilograms divided by the square of height expressed in meters (Kg/m^2^). Blood samples were collected in the morning after fasting overnight. Blood glucose, TC and TG were analyzed using enzymatic methods, whereas high-density lipoprotein (HDL)-cholesterol was measured after isolation of low-density lipoprotein (LDL), and LDL cholesterol was calculated using Friedewald’s formula.

### Statistical analysis

Data were normally distributed (evaluated by Kolmogorov-Smirnov test) and reported as mean ± standard deviation (SD) for quantitative variables, and count and percentage for categorical variables. Group differences were assessed using the unpaired Student’s t-test, one-way ANOVA with Tukey post-hoc tests, and Pearson’s Chi-squared (χ²) test, as appropriate. Pearson correlation analyses were conducted to identify relationships between variables and avoid multicollinearity issues. Multiple linear regression was used to explore associations between serum lipid levels and neuropsychological test scores, adjusting for identified confounding variables. Sample size estimation via post hoc linear regression showed a global effect size of 35%, type I error of 0.05, and 99% power (GPower 3.1.7). Two-tailed p-values ≤ 0.05 were considered significant. Analyses were conducted using SPSS 21 (SPSS, Inc., Chicago, IL).

## Results

### General descriptive analysis of sample characteristics

The sample population included 1283 subjects over 65 years, mainly women (*n* = 817; 63.6%), with a mean age of 79.79 ± 5.93 years. Demographic and clinic characteristics of the sample population stratified by CDR are shown in Table 1. Subjects with CDR ≥ 1 were older and had lower education, lower functional autonomy in ADL, lower functional autonomy in IADL, lower BMI, and lower systolic (SBP) and diastolic blood pressure (DBP), as compared to other groups. 475 (37%) of the sample population used anti-lipid drugs. 47 (3.6%) had a story of stroke, 58 (4.5%) suffered from myocardial infarction, 170 (13.2%) from type 2 diabetes, and 522 (40.6%) from hypertension. All subjects with diabetes and hypertension was under therapy. In CDR ≥ 1, there was a high percentage of women (67.4%). No other statistically significant differences were found among the variables examined.

**Table 1:** General characteristics of all sample population stratified by cognitive status (CDR) (*n* = 1283)

	Total (*n* = 1283)	CDR 0 (*n* = 247)	CDR 0.5 (*n* = 409)	CDR ≥ 1 (*n* = 627)	*p*
Sex (M/F)	466/817	94/153	168/241	204/423	**0.017**
Age (years)	79.79 ± 5.93	76.40 ± 6.30	78.97 ± 5.52	81.65 ± 5.30	**< 0.0001**
Education (years)	8.13 ± 4.81	11.26 ± 4.99	8.27 ± 4.63	6.81 ± 4.25	**< 0.0001**
ADL (n)	4.75 ± 1.41	5.47 ± 0.84	5.21 ± 0.97	4.17 ± 1.59	**< 0.0001**
IADL (n)	4.05 ± 2.63	6.42 ± 1.87	5.00 ± 2.25	2.50 ± 2.08	**< 0.0001**
BMI (kg/m^2^)	26.73 ± 4.58	27.73 ± 4.89	26.65 ± 4.28	26.36 ± 4.59	**< 0.0001**
SBP (mmHg)	130.89 ± 17.89	134.22 ± 20.23	132.15 ± 17.85	128.99 ± 16.82	**0.001**
DBP (mmHg)	73.37 ± 10.25	75.39 ± 10.54	74.11 ± 10.26	72.22 ± 10.00	**< 0.0001**
Glucose (mg/dl)	108.80 ± 33.94	106.31 ± 23.30	109.66 ± 32.48	109.06 ± 37.59	0.524
TC (mg/dl)	197.41 ± 45.09	199.35 ± 40.25	198.53 ± 44.00	196.03 ± 47.29	0.570
HDL-C (mg/dl)	56.96 ± 15.85	57.52 ± 16.87	57.51 ± 13.94	56.42 ± 16.66	0.515
LDL-C (mg/dl)	116.51 ± 37.27	116.85 ± 35.55	117.99 ± 37.34	115.45 ± 37.80	0.679
Triglycerides (mg/dl)	120.47 ± 56.96	119.78 ± 49.01	117.73 ± 58.06	122.49 ± 58.66	0.447
Stroke n (%)	47 (3.6)	6 (2.4)	13 (3.1)	28 (4.4)	0.274
Myocardial infarction n (%)	58 (4.5)	14 (5.6)	13 (3.1)	31 (4.9)	0.308
Type 2 Diabetes n (%)	170 (13.2)	29 (11.7)	49 (11.9)	92 (14.6)	0.321
Hypertension n (%)	522 (40.6)	119 (48.1)	163 (39.8)	240 (38.2)	0.060
Lipid drug use n (%)	475 (37.0)	90 (36.4)	154 (37.6)	231 (36.8)	0.944

CDR: Clinical Dementia Rating (CDR); ADL: Activities of Daily Living; IADL: Instrumental Activities of Daily Living; SBP: Systolic Blood Pressure; DBP: Diastolic Blood Pressure; TC: Total Cholesterol; HDL-C: High-Density Lipoprotein Cholesterol; LDL-C: Low-Density Lipoprotein Cholesterol. *p* by ANOVA or χ^2^ where appropriate

Tukey post hoc tests for multiple comparison

Height: CDR 0 vs. CDR 0.5 *p* = 0.201; CDR 0 vs. CDR ≥ 1 *p* < 0.0001; CDR0.5 vs. CDR 1 *p* < 0.0001

BMI: CDR 0 vs. CDR 0.5 *p* = 0.020; CDR 0 vs. CDR ≥ 1 *p* = 0.001; CDR0.5 vs. CDR 1 *p* = 0.652

SBP: CDR 0 vs. CDR 0.5 *p* = 0.408; CDR 0 vs. CDR ≥ 1 *p* = 0.001; CDR0.5 vs. CDR 1 *p* = 0.028

DBP: CDR 0 vs. CDR 0.5 *p* = 0.354; CDR 0 vs. CDR ≥ 1 *p* = 0.001; CDR0.5 vs. CDR 1 *p* = 0.020

Where do not explained, when ANOVA significant, all pairwise comparison among grooups by Tukey tests: *p* < 0.0001

Considering the aim of the study, and the sex disparity, the sample population was stratified by sex and demographic and clinic characteristics are showed in Table 2. Women had significantly lower years of education, lower functional autonomy in ADL, higher functional autonomy in IADL, lower glucose levels, higher TC, higher HDL-C levels, and higher LDL-C compared to men. Men were more likely to use lipid drugs, to have a story of myocardial infarction, and type 2 diabetes. Tables S2 and S3 show the clinical characteristics of the sample population stratified by the CDR separately in men and women. Men with CDR ≥ 1 were older, had lower years of education, lower BMI with a lower percentage of hypertension as compared with CDR 0.5 and CDR 0. No other statistically significant differences were found. Among women, subjects with CDR ≥ 1 were older, had lower years of education, had lower BMI, lower SBP, and lower DBP, as compared with CDR 0.5 and CDR 0 and healthy subjects. No other difference was found in other variables explored.

**Table 2:** Characteristics of all sample population stratified by sex (*n* = 1283)

	Women (*n* = 817)	Men (*n* = 466)	*p*
Age (years)	79.96 ± 5.91	79.49 ± 5.95	0.174
Education (years)	7.46 ± 4.56	9.31 ± 5.01	**< 0.0001**
ADL (n)	4.60 ± 1.45	5.01 ± 1.31	**< 0.0001**
IADL (n)	4.22 ± 2.78	3.75 ± 2.33	**< 0.0001**
BMI (kg/m^2^)	26.72 ± 4.89	26.74 ± 3.98	0.937
SBP (mmHg)	131.03 ± 17.48	130.65 ± 18.58	0.737
DBP (mmHg)	73.36 ± 10.33	73.39 ± 10.11	0.952
TC (mg/dl)	207.65 ± 43.60	179.71 ± 40.08	**< 0.0001**
HDL-C (mg/dl)	60.69 ± 15.50	50.54 ± 14.35	**< 0.0001**
LDL-C (mg/dl)	122.28 ± 36.03	106.52 ± 37.25	**< 0.0001**
Triglycerides (mg/dl)	120.09 ± 50.68	121.13 ± 66.50	0.767
Glucose (mg/dl)	106.72 ± 33.19	112.41 ± 34.94	**0.006**
Stroke n (%)	23 (2.8)	24 (5.1)	0.064
Myocardial infarction n (%)	24 (2.9)	34 (7.2)	**< 0.0001**
Type 2 Diabetes n (%)	90 (11.0)	80 (17.1)	**0.001**
Hypertension n (%)	338 (41.3)	184 (39.4)	0.549
Lipid drug use n (%)	286 (35.0)	189 (40.5)	**0.048**

ADL: Activities of Daily Living; IADL: Instrumental Activities of Daily Living; SBP: Systolic Blood Pressure; DBP: Diastolic Blood Pressure TC: Total Cholesterol; HDL-C: High-Density lipoprotein Cholesterol; LDL-C: Low-ensity Lipoprotein Cholesterol. *p* by unpaired t-Student or χ^2^ where appropriate

Thus, we performed simple linear correlation analyses between the variables that differed between groups and serum lipid levels. In women TC, LDL-C and HDL-C significantly correlated with years of education (*r* = 0.110, *p* = 0.003; *r* = 0.088, *p* = 0.043 and *r* = 0.116, *p* = 0.002 respectively), while TG significantly correlated with BMI (*r* = 0.201, *p* < 0.0001). In men serum TC and TG significantly correlated with years of education (*r* = 0.103, *p* = 0.036 and *r*=-0.104, *p* = 0.035) while TC also correlated with SBP (*r* = 0.157, *p* = 0.003) as well as TG with BMI (*r* = 0.249, *p* < 0.0001).

### Cognitive performances and serum lipid levels

Neuropsychological assessment in all sample population, stratified by CDR, is shown in Table 3. As expected across the population, subjects with CDR ≥ 1 had significantly lower performance in all neurocognitive areas explored compared to CDR 0.5 subjects and CDR 0 subjects.

**Table 3:** Neuropsychological assessment in all sample population, by cognitive status (CDR) (*n* = 1283)

	Total (*n* = 1283)	CDR 0 (*n* = 247)	CDR 0.5 (*n* = 409)	CDR 1 (*n* = 627)	*p*
**Screening**					
MMSE	22.23±6.13	28.80±1.45	25.22 ± 2.83	17.68 ± 5.22	**< 0.0001**
ACE-R	60.96±20.39	85.98±8.87	68.22 ± 12.04	45.27 ± 14.22	**< 0.0001**
ACE-R Attention/Orientation	13.79±3.88	17.51±0.86	15.61 ± 2.12	10.93 ± 3.49	**<0.0001**
ACE-R Memory	11.50±6.70	20.24±4.23	12.90 ± 5.15	6.84 ± 3.77	**< 0.0001**
ACE-R Language	18.95±6.02	24.36±2.83	21.01 ± 4.37	15.21 ± 5.56	**< 0.0001**
ACE-R Fluency	5.67±3.52	9.48±2.78	6.54 ± 2.71	3.45 ± 2.52	**< 0.0001**
ACE-R Visuo-spatial	11.00±3.68	14.36±1.94	12.11 ± 2.66	8.80 ± 3.43	**< 0.0001**
Clock Drawing Test	2.60±1.80	4.21±1.28	3.06 ± 1.68	1.61 ± 1.41	**< 0.0001**
**Attention**					
Attentional Matrices	33.88±13.49	46.37±8.60	36.75 ± 10.89	25.19 ± 11.46	**< 0.0001**
Trail Making Test A	131.58±135.95	58.41±29.11	98.68 ± 86.06	207.13 ± 172.83	**< 0.0001**
**Memory**					
Digit Span Forward	4.60±1.14	5.31±1.08	4.73 ± 0.95	4.14 ± 1.10	**< 0.0001**
Digit Span Backward	2.93±1.10	3.69±0.85	3.11 ± 0.94	2.39 ± 1.06	**< 0.0001**
Rey Auditory Verbal I	23.56±11.47	35.89±11.20	24.65 ± 8.81	16.06 ± 6.67	**< 0.0001**
Rey Auditory Verbal D	3.04±3.53	7.16±3.32	2.89 ± 3.01	0.99 ± 1.76	**< 0.0001**
Babcock story recall	3.18±3.69	9.36±4.08	4.00 ± 3.58	1.87 ± 2.54	**< 0.0001**
Corsi Span	3.78±1.21	4.46±0.94	4.00 ± 1.05	3.27 ± 1.25	**< 0.0001**
**Language**					
Letter fluency Test (FAS)	21.63±12.95	32.51±11.73	22.39 ± 11.40	14.43 ± 9.94	**<0.0001**
Categories fluency Test	12.07±6.55	19.28±6.71	11.73 ± 4.22	7.50 ± 3.78	**< 0.0001**
Token test	28.61±5.41	32.81±2.32	29.76 ± 4.52	25.44 ± 5.40	**< 0.0001**
**Executive Functions**					
Trail Making Test B	311.45±209.38	154.77±127.50	314.60 ± 204.77	426.20 ± 187.62	**< 0.0001**
**Fluid Intelligence and logic reasoning**					
Raven’s test	20.47±7.61	27.35±5.79	21.46 ± 5.93	15.72 ± 6.54	**< 0.0001**

CDR: Clinical Dementia Rating (CDR); MMSE: Mini-Mental State Examination; ACE-R: Addenbrooke’s Cognitive Examination. Rey Auditory Verbal I: Immediate recall, D: Delayed recall. *p* by ANOVA among the three groups. All pairwise comparison between groups by Tukey tests: *p* < 0.0001

In all population correlating serum lipid levels with all neuropsycological tests we found that TC significantly correlared with FAS (*r* = 0.136, *p* < 0.0001), RAVLT-I (*r* = 0.080, *p* = 0.019) and ACER-F (*r* = 0.069, *p* = 0.025). LDL-C correlated with FAS (*r* = 0.148, *p* < 0.0001), HDL-C with FAS (*r* = 0.112, *p* = 0.002), RAVLT-I (*r* = 0.072, *p* = 0.039) and ACER-F (*r* = 0.086, *p* = 0.006) while TG with ACER-F (*r*=-0.081, *p* = 0.009).

Thus, we conducted, in all population and separately for sex, multiple linear regression analyses to verify the association between serum lipid levels with all neuropsychological tests included in the study controlled for multiple identified confounding factors, including age, years of education, BMI, SBP, and lipid drug use (Table 4). No significant associations were found among the CDR 0 group in all population and in women, while a positive association between HDL-C and ACE-R Fluency in men (B: 0.058, β coefficient:0.314, standard error: 0.025, *p* = 0.026) was found. In the CDR 0.5 group, TC was associated with better performance in ACE-R Fluency (B: 0.008, β coefficient:0.124, standard error: 0.004, *p* = 0.039) as well as letter fluency test (FAS) (B: 0.032, β coefficient:0.129, standard error: 0.210, *p* = 0.039). HDL-C was associated with better performance in the letter fluency test (FAS) (B: 0.104, β coefficient:0.129, standard error: 0.048, *p* = 0.033). In men in the CDR 0.5 group, TC was associated with better performance in the TMT-B (B: -0.145, β coefficient: -0.265, standard error: 0.576, *p* = 0.013) and Token tests (B: 0.023, β coefficient:0.246, standard error: 0.010, *p* = 0.022). In women, CDR 0.5 a positive association was found between TC and RAVLT-I (B: 0.039, β coefficient:0.168, standard error: 0.015, *p* = 0.049). Considering the CDR ≥ 1 group, both TC (B: 0.034, β coefficient:0.161, standard error: 0.013, *p* = 0.012) and HDL-C (B: 0.090, β coefficient:0.150, standard error: 0.034, *p* = 0.009) were associated with better cognitive performance in the letter fluency test (FAS). In men, TC was associated with a worse cognitive performance in MMSE (B: -0.024, β coefficient: -0.203, standard error: 0.012, *p* = 0.044) and ACE-R Attention/Orientation (B: -0.019, β coefficient: -0.045, standard error: 0.077, *p* = 0.017). In women TC was associated with better cognitive performance in FAS (B: 0.035, β coefficient:0.0167, standard error: 0.016, *p* = 0.032) and ACE-R Fluency (B: 0.009, β coefficient:0.154, standard error: 0.004, *p* = 0.019).

**Table 4:** Significant multivariate linear regression models testing the association between lipid serum levels and cognitive scores

Model A	B; β coefficient (standard error); *R*^2^	*p* value
**All population CDR 0.5**	**ACER-F** **FAS**	
Total Cholesterol	0.008; 0.124 (0.004); 0.154 0.032; 0.129 (0.015); 0.210	0.039 0.039
	**FAS**	
HDL Cholesterol	0.104; 0.129 (0.048); 0.215	0.033
**All population CDR 1**	**FAS**	
Total Cholesterol	0.034; 0.161 (0.0133); 0.185	0.012
	**FAS**	
HDL Cholesterol	0.090; 0.150 (0.034); 0.207	0.009
**Model B**	**B; β coefficient (standard error); R** ^**2**^	***p*** **value**
**Men CDR 0**	**ACER-F**	
HDL Cholesterol	0.058; 0.314 (0.025); 0.268	0.026
**Men CDR 0.5**	**TMT-B** **Token**	
Total Cholesterol	-1.45; -0.265 (0.576); 0.242 0.023; 0.246 (0.010); 0.139	0.013 0.022
**Men CDR 1**	**MMSE** **ACER-O**	
Total Cholesterol	-0.024; -0.203 (0.012); 0.122 -0.019; -0.045 (0.077); 0.113	0.044 0.017
**Model C**	**B; β coefficient (standard error); R** ^**2**^	***p*** **value**
**Women CDR 0.5**	**RAVLT-I**	
Total Cholesterol	0.039; 0.168 (0.015); 0.132	0.049
**Women CDR 1**	**FAS** **ACER-F**	
Total Cholesterol	0.035; 0.167 (0.016); 0.183 0.009; 0.154 (0.004); 0.077	0.032 0.019

Model A tested after adjusting for age, sex, education, Body Mass Index (BMI), Systolic Blood Pressure (SBP), and lipid drug use; Model B and Model C adjusting for age, education, BMI, SBP, and lipid drug use

## Discussion

The relationship between lipid profiles and cognitive functions is still under debate in the old age population due to different and often contradictory findings. Some studies observed that lower TC levels correlated [17–19] with poorer cognitive performances in individuals over 60 [20, 21]. Conversely, others identified TC and LDL-C as risk factors for cognitive decline [22]. In contrast, others found no significant association between serum lipid levels and cognitive function in older adults [23, 24]. We hypothesized that these inconsistencies might be linked to differences in cognitive impairment stage and sex.

Our results indicate a significant positive association between total cholesterol (TC) and verbal fluency (ACER-F) in old individuals with MCI, independent of other factors. Additionally, both TC and HDL-C were associated to better language performance (FAS) in individuals with MCI or AD. These findings align with prior studies suggesting that hypercholesterolemia may protect against dementia and cognitive decline in those aged 75 and older [25]. Lower levels of naturally occurring total cholesterol are linked to worse cognitive performance among older persons aged 55 to 88 years in the Framingham Heart Study [26]. Additionally, a 26-year follow-up study among an old population found that serum cholesterol levels decreased in those who developed mild cognitive impairment while increased levels were found in those who preserved normal cognitive function [27]. Lower total cholesterol levels may be linked to reduced cognitive performance due to the critical role that plasma cholesterol plays in maintaining structural integrity and regulating the fluidity of neuronal cells [28]. Intriguingly, shortages in cellular cholesterol or its delivery to neurons have been found to hinder dendrite growth and synaptogenesis and promote neurodegeneration [29]. Furthermore, cholesterol is crucial for synapse formation in the central nervous system [30].

Cholesterol is crucial for various physiological functions, including the metabolism of steroid hormones and lipid-soluble vitamins, essential for synaptic integrity and neurotransmission. The link between higher cholesterol levels and better cognitive function aligns with findings that low cholesterol may weaken the blood-brain barrier, contributing to synaptic decline and cognitive deterioration. Additionally, the cholesterol-to-phospholipid ratio in cell membranes increases with age, leading to greater membrane rigidity [31, 32]. An in vitro study has suggested that cholesterol acts as an antioxidant and, therefore, has a protective role in the pathogenesis of dementia. Consistent with our findings, some previous studies have suggested the beneficial role of HDL-C in cognitive functions [33–35]. The ACER-F and FAS tests assess phonemic word fluency, a type of verbal fluency critical for retrieving information from memory, relying on executive functions like selective attention, mental shifting, and self-monitoring. HDL-C is vital for removing excess cholesterol from cells, transporting it to the liver, and protecting against atherosclerosis. Elevated levels of HDL-C have been associated with increased hippocampal volume and reduced amyloid-beta fibrillization [36, 37]. Additionally, HDL-C may act as a cardiovascular protector by facilitating reverse cholesterol transport and enhancing the delivery of apolipoprotein E (ApoE) [38, 39]. This protective function of HDL-C is important for preserving brain regions involved in verbal memory [34]. The Whitehall II study investigated HDL-C levels in relation to verbal memory deficits in middle-aged adults, highlighting the significance of HDL-C in cognitive health [40]. Notably, lower baseline HDL-C levels have been linked to reduced gray matter volumes in the temporal regions of the brain, which are critical for cognitive function. This connection underscores the positive impact of HDL-C on both cardiovascular health and cognitive performance outcomes.

However, several studies have indicated that the relationship between cholesterol and cognitive function may vary between older men and women. Thus, we performed the same regression analyses stratifying the population by sex. We found a positive association between HDL-C and ACER-F in cognitively healthy men. Again, in MCI, TC was associated with better performances in the TMT-B and Token test in men and with the RAVLT-I in women. The TMT assesses spatial planning ability in a visuomotor task and is extremely sensitive in detecting brain damage. Token Test aimed at detecting the residual potential for understanding spoken language. Rey’s auditory verbal learning test (RAVLT) is a well-known measure of episodic memory, and in previous studies, it has had a significant role in the early diagnosis of AD [41]. In subjects with dementia (CDR ≥ 1) higher TC levels are associated with a worse cognitive performance in the text of general cognition MMSE and ACER domains exploring orientation and attention in men. In contrast, in women, they are associated with better cognitive performance in verbal fluency (tested by FAS and ACER-F). These results suggest a different scenario. Only in old men, high levels of TC can be associated with a potentially higher risk for dementia, while in women, it might have a protective role, according to a previous finding [21]. It has been hypothesized that elevated levels of TC might promote cognitive decline by increasing the production and deposition of β-amyloid, as well as by fostering the formation of neurotoxic fibrils and neuritis, which could accelerate the progression of dementia [42, 43]. Sex differences in brain pathophysiology and dementia development have been reported in the literature [44, 45], and in all population studies that include cohorts of very older people, women represent a large part of the population. This sex disparity could stem from differences in lipid metabolism. Additionally, women tend to undergo changes in serum cholesterol levels at an earlier age and generally have higher cholesterol concentrations in later life than their male counterparts [46–48].

A key strength of this study is the large sample size, allowing analysis of the relationship between cholesterol levels, cognitive performance, and the effects of sex and cognitive impairment, while also accounting for lipid-lowering medications. However, its cross-sectional design limits causal conclusions, and it does not control for potential confounders such as biological factors, diet, or lifestyle habits. Furthermore, while we recorded whether participants with hypertension and diabetes were using medications, we did not have detailed data on the specific types of medications taken. However, this remains a limitation, as different classes of medications may have varying effects. Therefore, longitudinal or experimental studies are needed to confirm these findings and further explore the complex interactions between these variables.

### Conclusions and implications

In conclusion, higher late-life total cholesterol levels may protect cognitive performance in both cognitively healthy individuals and those with mild cognitive impairment. This association remains significant in women with dementia but not in men, indicating a sex-specific effect. Understanding this relationship may aid in preventing cognitive decline, suggesting the need for tailored strategies for men and women with dementia in future research.

## Data Availability

The data that support the findings of this study are not openly available (due to reasons of privacy) but are available from the corresponding author upon reasonable request.
